# Assessment of the Effectiveness of Team-based Learning Activities on Learning Outcomes in the Undergraduate Immunology Classroom

**DOI:** 10.4049/immunohorizons.2300073

**Published:** 2024-01-22

**Authors:** Joshua J. Baty, Heather A. Bruns

**Affiliations:** Department of Microbiology, University of Alabama at Birmingham Heersink School of Medicine, Birmingham, AL

## Abstract

Immunology is inherently interdisciplinary. Understanding how the immune system functions requires knowledge from several scientific disciplines, including molecular biology, cellular biology, genetics, and biochemistry. Furthermore, immunology is conceptually complex, requiring the identification of a plethora of immune components and mastery of a large volume of new vocabulary. These attributes can pose challenges to student learning in the undergraduate immunology classroom. Team-based learning (TBL) is a pedagogical method used to increase student engagement in learning, improve student collaboration, and develop communication skills. In a variety of educational settings, TBL activities have been shown to foster a deeper understanding of complex topics, increase student confidence in course content, and improve learning outcomes. In this study, we examined differences in the impact of traditional lecture versus TBL activities on student learning outcomes for four different topics presented in an undergraduate adaptive immunity course composed largely of academically high-performing students. We matched content across two student cohorts, delivered via team-based learning methodology (T cell development and Ab-mediated functions) and traditional lecture (B cell development and T cell effector functions). Student learning was assessed using content questions across a range of Bloom’s taxonomy levels, which demonstrated that the TBL activities did not improve examination performance over lecture-based learning in this course. However, students found this learning tool to be valuable, indicating that the TBL activities assisted with preparation for examinations and provided a necessary opportunity to address misconceptions.

## Introduction

Immunology is a complex and rapidly evolving field. It can be engaging for students because of its connection to their own personal health, its importance in the biomedical field, and its application of information learned in other science courses. However, immunology can also be challenging because of its requirement for integrated knowledge from several different scientific disciplines, such as biochemistry, cellular biology, genetics, anatomy, and physiology. Students often struggle with the vast amount of terminology and specialized terminology of the discipline, which can be technically confusing (1). Furthermore, there are few resources available to immunology educators to aid in targeted selection of content and effective pedagogical practices in the undergraduate immunology classroom (2–4), and there is only moderate consensus among immunology educators on critical topics that should be covered (5).

Identifying content to present to students in the undergraduate immunology classroom and the methods used to present that content can be critical determinants of student success in the course and increased interest and engagement in the field. Instructor-centered, lecture-based learning (LBL) is the most prevalent pedagogical approach in the STEM (science, technology, engineering, and mathematics) classroom (6), despite being less effective than student-centered strategies at engaging students and improving learning outcomes (7). Team-based learning (TBL) is an active-learning, student-centered teaching method that is based on constructivist learning theory (8) and developed to improve learning outcomes, particularly in courses that cover a large amount of information that is also to be applied to answer complex questions or solve significant problems. TBL requires that students do preparation ahead of class, for which they are held accountable by an individual readiness assessment test (iRAT) that is subsequently discussed in groups. Following discussion of the iRAT, the group submits a team/group readiness assessment test (gRAT). Following the gRAT, application questions are posed to the groups and discussed as a class. Finally, students submit peer evaluations (9, 10).

TBL has been shown to improve several learning outcomes as assessed through course grades, knowledge retention, and examination performance in a variety of science disciplines: anatomy, biology, biochemistry, and psychology (11–14). The benefits of using the TBL method to teach immunology and integrate foundational and clinical concepts in the medical curriculum have been well documented (15–18). However, there have been few investigations into the effectiveness of TBL in the nonclinical undergraduate immunology classroom.

The goal of this study was to evaluate differences in presenting immunology content using flipped-classroom TBL activities or by traditional lecture on learning outcomes and student perceptions of learning and development of teamwork skills in an undergraduate adaptive immunity course.

## Materials and Methods

### Participants and learning activity design

This study was conducted in MIC 402, Adaptive Immunity, the third course in the core sequence of the undergraduate immunology major at the University of Alabama at Birmingham. Students taking this course are juniors in the Undergraduate Immunology Program at the University of Alabama at Birmingham. Approximately 70% of students in the Undergraduate Immunology Program are also in the Honors College. In the spring of 2022, 28 students were enrolled in the course, and in 2023, 30 students were enrolled in the course. This study and its procedures were reviewed and approved by the University of Alabama at Birmingham Institutional Review Board (protocol 300008830).

The MIC 402 course is taught using a variety of teaching methods in addition to traditional LBL, including informal group work to analyze data and discuss journal articles, as well as more formal group work in the form of TBL. For this study, four class sessions were identified as having related content and similar difficulty: B cell development, T cell development, T cell effector functions, and Ab-mediated functions. Two class sessions were delivered by lecture (B cell development and T cell effector functions), and two sessions were delivered by a flipped classroom TBL method (T cell development and Ab-mediated functions). The TBL platform, InteDashboard (CognaLearn Pte., Singapore), was used for all TBL activities. For the TBL activity, lectures on the content were created and recorded. Students were required to watch the lecture video prior to class, review the notes, and, because of time constraints in the class session, complete the iRAT individually online using InteDashboard before coming to class. Each iRAT consisted of 10 questions based on the required prework. At the start of class, students, in teams, completed the gRAT, which included the same questions as the iRAT, which allowed individuals to reason through any unclear questions with peers. Teams were created and balanced on the basis of performance and participation in MIC 401, Innate Immunity, in the prior semester. After clarifying any content assessed in the RAT, teams applied the information to complete application exercises. Application exercises were focused on clinical topics and scientific literacy skills and designed for group discussion to foster teamwork skills. Because of time constraints for each class session, the peer evaluation portion of the TBL was not done. In the prior semester, students had worked in teams for other TBL and group activities. Because of time constraints in that semester, a classroom policy of communicating group issues to the instructor had been well established. Thus, although formal peer evaluation would have been preferred as part of the TBL, students were well informed and used the alternative classroom practice when issues arose.

### Assessment data

B cell development (LBL) and T cell development (TBL) sessions were delivered as part of the content for Exam 1. T cell effector (LBL) and Ab-mediated (TBL) functions were delivered as part of the content for Exam 2. LBL and TBL class sessions were done on back-to-back class days, and examinations were administered within 2 wk of the content delivery. For both examinations, four multiple choice questions were written that assessed understanding of the main topics for each LBL and TBL session. Questions for each LBL and TBL content set (i.e., T and B cell development) were developed and matched according to Bloom’s taxonomy (Supplement 1). Only four questions per class session were specifically developed, because we had prior examination questions that had been used since the inception of the course that allowed us to look at data that were matched over time compared with these cohorts specifically. Thus, relevant examination questions from Exams 1 and 2 from four academic years (2020–2023) were also evaluated. Student performance on iRAT and gRAT assessments were evaluated for the 2022 and 2023 cohorts as well as course grades.

Course evaluation was composed of three examinations (100 points each), one cumulative final (100 points), three TBL sessions (25 points), critical review of a scientific article (individual assignment, 35 points), and a group presentation (50 points).

### Survey instrument

A survey was developed using Qualtrics (Seattle, WA) to gather data on students’ perceptions of learning, development of teamwork skills, and effectiveness of TBL. The survey was distributed to students as a link sent via e-mail or by QR code displayed at the end of a class session. The survey was anonymous, and participation was voluntary. The response rate was 79% (22 of 28) for the 2022 cohort and 70% (21 of 30) for the 2023 cohort.

### Data analysis

Student learning was assessed by the following comparisons: (1) matched Bloom’s taxonomy and topic, (2) iRAT versus gRAT performance, (3) assessing the outcomes from the exact same questions across cohorts where content was taught via LBL for 2 y and TBL for 2 y, and (4) examination questions on matched LBL and TBL content. Normality was assessed using the Shapiro–Wilk test. All comparisons were performed using paired *t* rests or one-way ANOVAs (nonparametric analyses where indicated). Correlation analyses were performed where indicated. All statistics were performed using GraphPad Prism (version 9.2.0 for Windows; GraphPad Software, La Jolla, CA).

## Results

**FIGURE 1.**
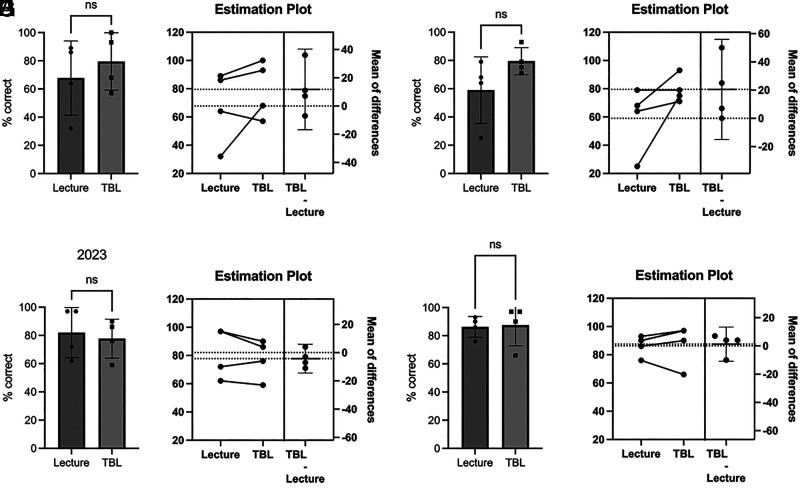
TBL did not improve content knowledge compared with LBL. Student performance on four multiple choice questions for each content topic (eight total per examination) matched by content similarity was evaluated across two academic years, 2022 (**A**–**D**) and 2023 (**E**–**H**). *n* = 4, *p* > 0.05, ns (paired *t* test, *n* = 4). LBL topics: B cell development (A, B, E, F) and T cell functions (C, D, G, H). TBL topics: T cell development (A, B, E, F), Ab-mediated functions (C, D, G, H).

To investigate the effect of TBL compared with LBL on learning outcomes, four immunology topics of related content with similar difficulty were delivered to students via the TBL method (T cell development and Ab-mediated functions) or by traditional lecture (B cell development and T cell effector functions). Student learning was assessed using topic and Bloom’s taxonomy matched examination questions. For these student cohorts, examination question means demonstrated that TBL did not significantly improve learning outcomes compared with LBL (Fig. 1). Although not significant, the 2022 cohort demonstrated an increase in the mean scores of examination questions for topics taught by TBL, T cell development and Ab-mediated functions, compared with B cell development and T cell effector functions (Fig. 1A, 1C), suggesting that TBL may have aided in learning more than LBL. However, this was not observed for the 2023 cohort (Fig. 1E, 1G), which had overall higher performances on examination questions on both topics.

**FIGURE 2.**
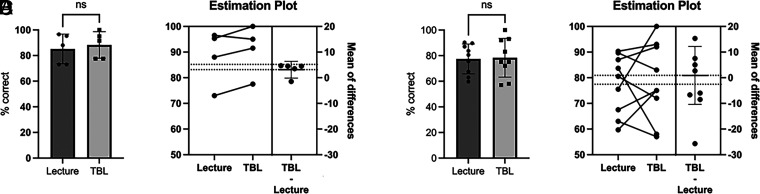
TBL activities do not increase gains in content knowledge, regardless of Bloom’s taxonomy designation. Outcomes on matched content were compared between LBL and TBL. Content was divided into lower-order (**A** and **B**) and higher-order (**C** and **D**) Bloom’s taxonomy. Paired *t* test (A), Wilcoxon matched-pairs signed-rank test (C) *n* = 5–9. ns, *p* > 0.05.

To evaluate if TBL, compared with LBL, improved student performance requiring higher-order versus lower-order thinking skills, student performance was evaluated for content-matched examination questions across 4 y (including the eight examination questions analyzed in Fig. 1) that were categorized into lower-order (remembering) and higher-order (understanding) Bloom’s taxonomy (Fig. 2). Differences in examination question means between lower-order (Fig. 2A, 2B) and higher-order (Fig. 2C, 2D) Bloom’s taxonomy demonstrated that TBL did not increase gains in content knowledge above LBL.

**FIGURE 3.**
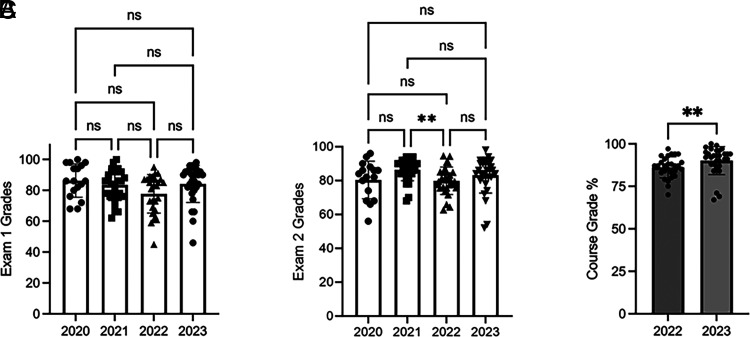
Evaluation of examination scores and course grades among cohorts. Student performance on Exams 1 (**A**) and 2 (**B**) was evaluated across four academic years. Examination scores were assessed using one-way ANOVA (Exam 1) and Kruskal–Wallis analysis (Exam 2), followed by a Tukey’s post hoc test. ns, *p* > 0.05, **p < 0.01. Course grades (**C**) were evaluated by a Mann–Whitney test, *n* = 28–30, **p < 0.01.

Although there were no significant differences in the effects of TBL on learning (Figs. 1 and 2), there was a trend toward improved learning in the 2022 cohort. To assess differences in cohorts, course grades for the past 2 y were evaluated. Additionally, examination scores across four academic years on the same content taught via TBL or LBL, depending on the year, were evaluated. Student performance on both Exam 1 (Fig. 3A) and Exam 2 (Fig. 3B) were similar. Although the 2022 cohort performed significantly less well than the 2021 cohort on examination 2, there was no significant difference between the 2022 and 2023 cohorts in overall examination performance. However, overall course grades for the 2023 cohort were higher than those of the 2022 cohort (Fig. 3C).

**FIGURE 4.**
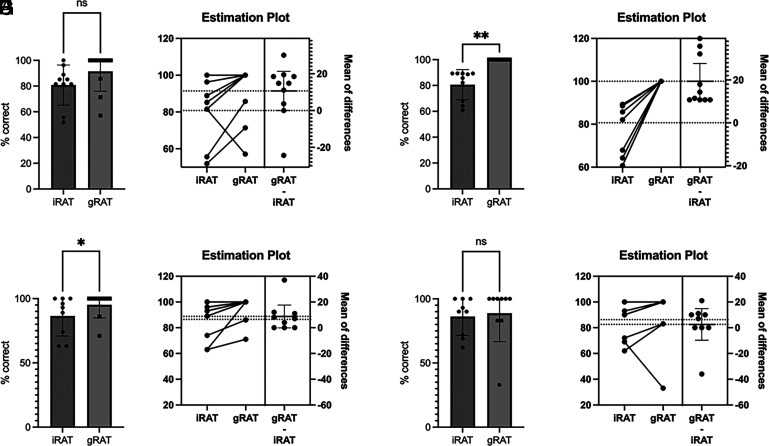
TBL assessments demonstrate short-term gains in learning. iRAT and gRAT performance for the T cell development (**A**–**D**) and Ab-mediated functions. (**E**–**H**) TBLs were evaluated for the 2022 (A, B, E, F) and 2023 (C, D, G, H) cohorts. Wilcoxon test (A, C, E, G), *n* = 8–10; *p < 0.05, **p < 0.01.

**FIGURE 5.**
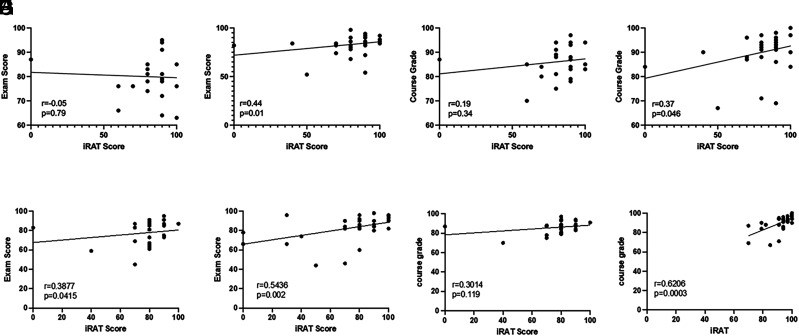
iRAT performance as an indicator of both examination and course performance. The correlation of iRAT performance with examination (**A**, **B**, **E**, **F**) and course (**C**, **D**, **G**, **H**) grades for both the 2022 (A, C, E, G) and 2023 (B, D, F, H) cohorts was evaluated for both the Ab-mediated functions TBL (A–D) and the T cell development TBL (E–H). Pearson correlation (A, C, G, H); Spearman correlation (B, D, E, F). *n* = 28–30.

Although TBL did not improve gains in knowledge acquisition above that of LBL in this study, TBL activities increased other measures of learning. Comparing the 2022 and 2023 cohort performance on the iRAT and gRAT assessments, we found that both cohorts demonstrated short-term gains from individual to team performance, although this was only demonstrated for one TBL session, and it was not the same TBL for each cohort (Fig. 4C–4F). Furthermore, iRAT performance was a significant indicator of both examination and course performance in the 2023 cohort, demonstrating positive correlations between iRAT scores and performance on both examinations and overall course grade (Fig. 5B, 5D, 5F, 5H), which was not observed in the 2022 cohort with the exception of examination performance for the T cell development TBL (Fig. 5A, 5C, 5E, 5G). Conversely, there was no correlation between gRAT scores and examination or course performance (data not shown).

**FIGURE 6.**
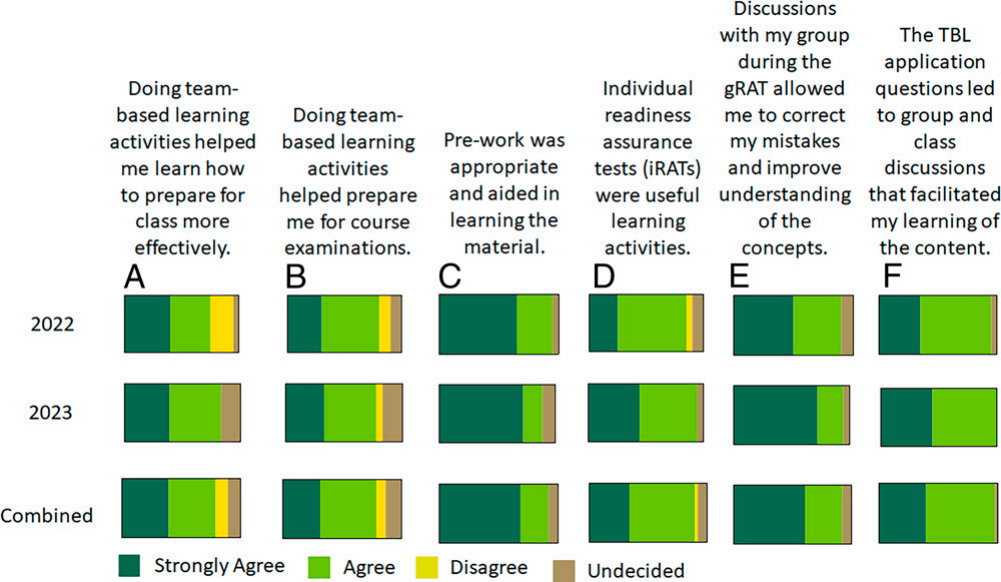
Assessment of student perceptions of the effectiveness of TBL on learning. Responses from survey questions (**A**–**F**) reported using a 5-point Likert scale are depicted as parts of the whole.

**FIGURE 7.**
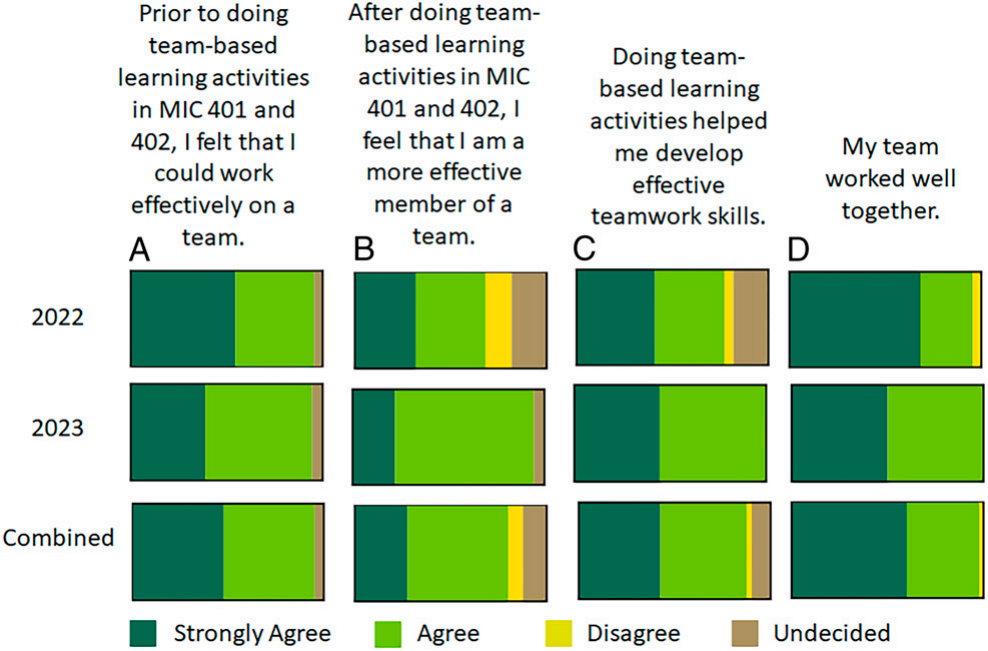
Assessment of student perceptions of the effectiveness of TBL on developing teamwork skills. Responses from survey questions (**A**–**D**) reported using a 5-point Likert scale are depicted as parts of the whole.

Student perceptions of the effectiveness of TBL on learning and development of teamwork skills were also assessed. A majority (>75%) of respondents in both cohorts reported in the affirmative that doing TBL activities helped them prepare for class sessions and examinations (Fig. 6A, 6B). Furthermore, greater than 90% of survey respondents in both cohorts reported that group discussions improved their understanding of concepts (Fig. 6E) and that TBL application questions facilitated their ability to learn the content (Fig. 6F). With regard to TBL activities enhancing the development of teamwork skills, ∼95% of survey respondents in both cohorts reported that they could work productively on a team prior to doing any TBLs in the course (Fig. 7A). Greater than 90% of the survey respondents in the 2023 cohort also reported that they felt like a more effective member of a team after doing the TBL activities in the course (Fig. 7B), but only 68% of the survey respondents in the 2022 cohort reported a perceived positive improvement in being an effective team member, with 14% disagreeing and 18% unsure about the effect of TBL activities on their effectiveness as a team member. Similar differences in student perceptions between cohorts were also observed when asked if TBL activities helped them develop effective teamwork skills (Fig. 7C).

## Discussion

The findings from this study demonstrated that TBL activities implemented in a small adaptive immunity course composed largely of high-performing students did not improve learning outcomes, specifically content knowledge, compared with LBL (Fig. 1) as assessed with both lower- and higher-order Bloom’s taxonomy level questions (Fig. 2). However, iRAT performance was positively correlated with both examination performance and overall course performance for the 2023 cohort, indicating that individual preparedness is a large predictor of student success. Although a majority of respondents in both cohorts regarded the TBL activities favorably (Fig. 6), fewer students in the 2022 cohort perceived the TBL activities as enhancing their teamwork skills or effectiveness as a team member (Fig. 7).

The positive effects of TBL, such as increased engagement, greater satisfaction with the course, heightened interest, and in particular content knowledge (19, 20), are well documented (21–23) in a variety of courses and classrooms. Additionally, a recent systematic review of studies evaluating the impact of TBL on learning outcomes in a diverse array of health care professional curricula reported that ∼66% of studies demonstrated improved academic performance compared with LBL (24). Consistent with our findings, however, other studies have reported that TBL activities do not always improve content knowledge, even if the activities are perceived as valuable by the students. Although increased engagement and integration of clinical and basic science knowledge were positively perceived by students because of TBL, examination performance did not demonstrate increased knowledge acquisition in a neuroanatomy course (25). Similar outcomes were observed in a medical gross anatomy and embryology course (26). It is important to note that the interpretation of the findings from this study do not suggest that TBL is ineffective; rather, it highlights the value in assessing the effectiveness of a chosen pedagogical approach in a given classroom setting and for a specific student population. Importantly, for academically lower-performing students in the preclinical medical curriculum, TBL can improve learning outcomes, such as examination performance, and decrease course failures to a greater extent than for higher-performing students (20, 27, 28). Students in our undergraduate course of study are majors in immunology, and ∼70% of students in the major are also accepted into programs within the Honors College. Thus, a large majority of students in these cohorts are high academic achievers. Indeed, previous studies have demonstrated that academically high-performing students tend to have increased motivation and can effectively acquire content knowledge through a variety of methods (29, 30). Thus, tailoring the educational intervention to the specific student population is an important consideration.

Notably, the few significant findings in this study were observed differently in each cohort: short-term learning gains as demonstrated by iRAT and gRAT performance for a different TBL in each cohort (Fig. 4), correlations between iRAT and examination/course performance (Fig. 5), and overall course grades (Fig. 3C), such that the 2022 cohort demonstrated slightly lower performance compared with the 2023 cohort. Although differences exist between cohorts in any academic program, the 2022 cohort was largely impacted by the COVID-19 pandemic. This cohort shifted to online learning in the spring of their freshman year, which continued into their sophomore year. The negative impacts on learning across academic levels due to the pandemic and subsequent shift to online learning are documented and continue to be evaluated (31–34). Although not significant, there was evidence to suggest that content delivery by TBL somewhat improved examination performance for the 2022 cohort (Fig. 1), which was not observed in the 2023 cohort (Fig. 2). Although a variety of factors may be responsible for this outcome, these data may suggest that TBLs are more effective in student groups whose education has been negatively impacted by the COVID-19 pandemic. Difficulty in maintaining student engagement in online learning has been well documented (35, 36), so the introduction of TBLs for these cohorts may increase engagement as well as learning.

There are limitations to the conclusions drawn from our investigation: notably, the small sample size and limited question bank. Ultimately, the goal of this study was to evaluate if the teaching methods used in this specific course within our program were beneficial enough to the students to warrant instructor time in development and continued use instead of other team- or group-based modalities. Although sufficient sample size is certainly important, particularly when considering the value of the conclusions that can be extrapolated from a study, there is value in small sample research, especially in education, to identify effective methods within a specific academic setting (37). Similar considerations were made regarding the choice of assessment and the limited (four questions per class topic) creation of new examination questions. Four questions per class session were created because we included other examination questions that had been used since the inception of the course, which allowed us to evaluate data that were matched over time (Fig. 2) compared with these cohorts specifically.

Taken together, our data indicate that although undergraduate immunology students perceive TBL favorably and some mild gains in content knowledge due to TBL activities were seen, TBL did not significantly improve student performance. Despite that, this study elucidated differences between our 2022 and 2023 cohorts that may have been exacerbated by the COVID-19 pandemic. Because teaching methods should be routinely tailored to student cohorts over time, it is important to evaluate content and pedagogy regularly. Furthermore, undergraduate science classrooms employing universal design likely benefit their students through teaching in a variety of ways, including through TBL—especially for challenging or detailed topics where group and class discussion may ease learning. More research is needed to fully understand the effectiveness of TBL in different science disciplines and educational contexts. Overall, the effectiveness of TBL in the science classroom may depend on a variety of factors, including the specific subject matter, the structure of the course, and the quality of the instructional design.

## Supplementary Material

Supplemental 1 (PDF)
