# Supplementary material for: Assessment of the Effectiveness of Team-based Learning Activities on Learning Outcomes in the Undergraduate Immunology Classroom
Source: Immunohorizons. 2024 Jan 22;8(1):106–13. doi: 10.4049/immunohorizons.2300073 (PMC10835648; doi:10.4049/immunohorizons.2300073)
Supplement: Supplemental 1 (PDF) [file IH_2300073_Supplemental_1.pdf]

| | | | |
|---|---|---|---|
| **Exam 1 Topic** | **B cell development** | **T cell development** | **Bloom's level** |
| Q1 | Which of the following are critical factors for B cell development?<br>*a. CXCL12, SCF, and IL7.<br>b. CXC13, IL4, and Notch<br>c. S1P, IL6, and TSLP<br>d. GCSF, GMCSF, IL13 | Which of the following is a critical factor for T cell fate determination?<br>a. The EBF transcription factor<br>*b. Notch<br>c. TLR9<br>d. ERBA | Remember |
| Q2 | What is the purpose of B cell development checkpoints?<br>*a. To test for the rearrangement of the heavy and light chain genes.<br>b. To initiate the B cell lineage commitment program.<br>c. Ensure the negative selection of auto-reactive B cells.<br>d. To induce switching from IgM to IgG isotypes. | What is the purpose of positive selection during T cell development?<br>a. To test for the rearrangement of the beta and alpha chains.<br>*b. To ensure TCRs recognize host MHC molecules.<br>c. To induce commitment to the T cell lineage.<br>d. To induce the expression of both CD4 and CD8. | Understand |
| Q3 | Which of the following BEST describes how B cell selection against self-antigen occurs?<br>a. Cells that experience low levels of auto reactive signaling are recruited into a specialized compartment called the marginal zone.<br>b. Selection is mediated by specialized epithelial cells in the bone marrow that present proteins from several host tissues.<br>*c. BCR binding of auto antigen in the spleen results in death of immature B cells.<br>d. Selection is carried out before commitment to the B cell lineage. | Which of the following BEST describes how T cell selection against self-antigen occurs?<br>*a. Selection is mediated by specialized epithelial cells that present proteins from organs other than the thymus.<br>b. TCRs engage antigen presented by professional antigen presenting cells in the spleen.<br>c. Selection is carried out in the bone marrow before T cell commitment.<br>d. Selection is mediated by TCR interactions with MHC molecules on thymic dendritic cells. | Understand |
| Q4 | A population of developing B cells is isolated from the bone marrow to be used in an experiment examining B cell development. The investigator only wants to use B cells that are at a specific stage of development. Based on the flow data presented below, which is examining the expression of lambda5, the cells in the box with the "24" are at which stage of development? | An investigator has performed flow cytometry to identify the percentages of thymocytes in different stages of development. Based on the flow cytometry data represented below, what process is occurring in the thymocyte population identified by the arrow on the dot plot? | Analyze |

| | | | |
|---|---|---|---|
| | 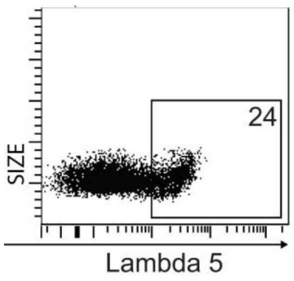<br>a. Pro-B<br>b. *Pre-B<br>c. Immature<br>d. Mature | 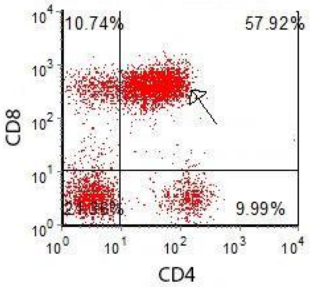<br>a. Beta chain rearrangement<br>b. Alpha chain rearrangement<br>c. Negative selection<br>d. *Positive selection | |
| | | | |
| **Exam 2 Topic** | **Antibody-mediated functions** | **T cell effector functions** | **Bloom's level** |
| Q1 | Which isotype will a TH2 polarized response will induce?<br>a. IgG2a antibody isotypes in mice.<br>b. IgM responses in mice.<br>c. IgA responses in mice.<br>*d. IgE responses in mice. | The differentiation of which CD4+ T cell subset will be induced in the presence of IL-2 and IFNgamma?<br>*a. Th1<br>b. Th2<br>c. Tfh<br>d. Treg | Remember |
| Q2 | Based on the expression of a variety of Fc receptors on several different cells, which of the following antibody isotypes has the GREATEST ability to regulate antibody-mediated functions?<br>a. IgM<br>*b. IgG<br>c. IgE<br>d. IgA | In addition to presentation of peptide in MHC Class I by an antigen presenting cell, which CD4+ T cell subset is necessary for the activation of CD8+ T cells?<br>a. Tfh<br>b. Th17<br>c. Th2<br>*d. Th1 | Understand |
| Q3 | Which of the following statements about antibodies of different isotypes is the MOST accurate?<br>a. They never share the same specificity.<br>b. They are present in the body in approximately equal amounts.<br>*c. They have unique effector functions.<br>d. They all interact with complement proteins. | Which of the following statements about cytotoxic T cells is the MOST accurate?<br>a. All activated cytotoxic T cells live for several months<br>b. Regulatory T cells do not influence the activation of cytotoxic T cells<br>*c. Cytotoxic T cells may use more than one mechanism to kill target cells<br>d. Cytotoxic T cells only form immunologic synapses with professional antigen presenting cells | Understand |
| Q4 | Which of the following BEST describes the purpose of antibody class switching?<br>a. to ensure that all varieties of pathogens | Which of the following BEST describes the purpose of CD4+ T cell differentiation?<br>a. to ensure that multiple classes of | Analyze |

| | | | |
|---|---|---|---|
| | can be neutralized<br>*b. to ensure that a variety of antibody-stimulated functions can be activated in response to a pathogen<br>c. to ensure that plasma cells are generated<br>d. to ensure that germinal centers are properly formed | antibodies are produced<br>b. to ensure that CD8+ T cells are properly activated<br>*c. to ensure an effective immune response is specifically mounted to an invading pathogen<br>d. to ensure immune responses are properly activated | |

Discrimination and % correct for both years that the exam questions were used.

| | **B cell dev (lecture)** | | | | **T cell dev (TBL)** | | | |
|---|---|---|---|---|---|---|---|---|
| | 2022 | | 2023 | | 2022 | | 2023 | |
| | % correct | disc | % correct | disc | % correct | disc | % correct | disc |
| Q1 | 89 | 0.23 | 97 | 0.39 | 100 | 0 | 90 | 0.58 |
| Q2 | 86 | 0.29 | 97 | 0.61 | 93 | 0.06 | 86 | 0.34 |
| Q3 | 32 | 0.41 | 72 | 0.45 | 68 | 0.8 | 76 | 0.4 |
| Q4 | 64 | 0.35 | 62 | 0.54 | 57 | -0.02 | 59 | 0.28 |
| | | | | | | | | |
| | **T cell eff fxns (lecture)** | | | | **Ab-med fxns (TBL)** | | | |
| | 2022 | | 2023 | | 2022 | | 2023 | |
| | % correct | disc | % correct | disc | % correct | disc | % correct | disc |
| Q1 | 64 | 0.58 | 86 | -0.13 | 71 | 0.55 | 90 | 0.42 |
| Q2 | 25 | 0.52 | 76 | 0.16 | 75 | 0.27 | 66 | 0.17 |
| Q3 | 68 | 0.41 | 93 | 0.44 | 93 | 0.32 | 97 | 0.66 |
| Q4 | 79 | 0.05 | 90 | 0.22 | 79 | -0.01 | 100 | 0 |
